# First-Row Transition 7-Oxo-5-phenyl-1,2,4-triazolo[1,5-a]pyrimidine Metal Complexes: Antiparasitic Activity and Release Studies

**DOI:** 10.3390/ph16101380

**Published:** 2023-09-28

**Authors:** Álvaro Martín-Montes, Sandra Jimenez-Falcao, Santiago Gómez-Ruiz, Clotilde Marín, José M. Mendez-Arriaga

**Affiliations:** 1 Departamento De Parasitología, Universidad De Granada, Avenida Fuentenueva, 18071 Granada, Spain; amartim@ugr.es; 2 Organic Nanotechnology Lab, Departamento De Materiales Y Producción Aeroespacial E.T.S.I Aeronáutica Y Del Espacio, Universidad Politécnica De Madrid, 28040 Madrid, Spain; sandra.jfalcao@upm.es; 3 COMET-NANO Group, Departamento De Biología y Geología, Física Y Química Inorgánica, E.S.C.E.T., Universidad Rey Juan Carlos, Calle Tulipán s/n, 28933 Móstoles, Spain; santiago.gomez@urjc.es

**Keywords:** metal complex, coordination chemistry, leishmaniasis, antiparasitic activity, drug release, nanoparticles

## Abstract

Leishmaniasis and Chagas disease are still considered neglected illnesses due to the lack of investment in research, despite the fact that almost one million new cases are reported every year. Four 7-oxo-5-phenyl-1,2,4-triazolo[1,5-a]pyrimidine (HftpO) first-row transition complexes (Cu, Co, Ni, Zn) have been studied for the first time in vitro against five different species of *Leishmania* spp. (*L. infantum*, *L. braziliensis*, *L. donovani*, *L. peruviana* and *L. mexicana*) as well as *Trypanosoma cruzi*, showing higher efficacy than the reference commercial drugs. UV and luminescence properties were also evaluated. As a proof of concept, anchoring of a model high-effective-metal complex as an antiparasitic agent on silica nanoparticles was carried out for the first time, and drug-release behaviour was evaluated, assessing this new approach for drug vehiculation.

## 1. Introduction

Triazolopyrimidine derivatives have been known since the beginning of the last century, when Bülow and Haas published for the first time the synthesis of some of them [1]. Nevertheless, their complexation properties were not studied until 1952, by Birr [2], who used this organic family to stabilize photographic silver emulsions. Since then, applications, including antipyretic, analgesic, anti-inflammatory, herbicidal, antifungal, antimicrobial, antitumor and antiparasitic properties [3,4], have been displayed for triazolopyrimidine ligands, as well as for their metal complexes, because of their remarkable pharmacology [5]. In the specific case of 1,2,4-triazolo[1,5-a]pyrimidines ligands, their structural similarity with purines makes them excellent candidates for the study of reactivity with different metal ions as biomimetic models [6,7,8,9,10], due to their structural similarity with these nucleobases. Triazolopyrimidines exhibit great versatility as ligands, owing to the presence of accessible electron pairs on their skeleton, which are located in at least three nitrogen atoms. Their main coordination mode is via N3-monodentate or N3,N4-bridging, but many other bonding routes could also be possible when the number of exocyclic substituents in the aromatic ring increases. The interaction between metal ions and 1,2,4-triazolo[1,5-a]pyrimidines ligands shows a great capability to act as building blocks with great versatility, with applications for the synthesis of metal–organic frameworks (MOFs) or multidimensional systems with useful properties such as magnetism, luminescence or biological activity [11,12,13,14,15].

**Scheme 1:**
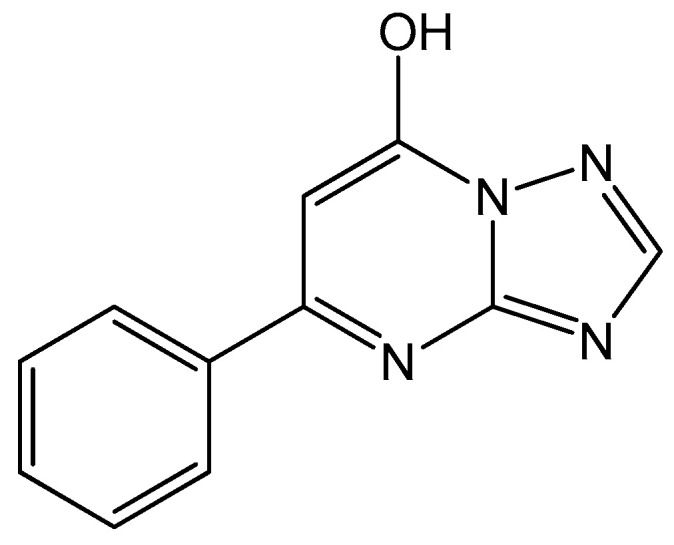
**HftpO** derivative structure.

The triazolopyrimidine family mentioned before includes a wide variety of derived structures, such as the 1,2,4-triazolo[1,5-a]pyrimidine itself (**tp**) in its non-substituted form [16] and the 5,7-dimethyl-1,2,4-triazolo[1,5-a]pyrimidine (**dmtp**) [17] and 7-amine-1,2,4-triazolo[1,5-a]pyrimidine (**7-atp**) [18] structures. Additionally, among the most reactive groups of triazolopyrimidines are those containing exocyclic oxygen atoms. These additional oxygen coordination positions spread out the range of metallic centres that can interact with these ligands (i.e., lanthanide ions). Some examples of oxygenated triazolopyrimidine derivatives are dihydro-5-oxo-[1,2,4]triazolo-[1,5-a]pyrimidine (**5HtpO** and **7HtpO**), whose structures were described by Abul Haj et al. [19], or the commercial 4,7-dihydro-5-methyl-7-oxo[1,2,4]-triazolo[1,5-a]pyrimidine (**HmtpO**) [20]. One of the last-synthesized and characterized oxo triazolopyrimidine derivatives, 7-oxo-5-phenyl-1,2,4-triazolo[1,5-a]pyrimidine (**HftpO**) [21] (Scheme 1), has been employed in the present study.

Leishmaniasis is one of the seven primary illnesses that are present in all continents. According to the World Health Organisation (WHO), it is endemic nowadays in almost 100 countries, with more than 350 million people on the planet at risk to be infected. Millions of new cases appear every year, and the mortality rate is higher than 60,000 deaths in a year, a value among parasitic diseases which is only surpassed by malaria. Although it is usually located in poor and unhealthy areas of the Third World, it is also present in Europe and Asia due to migration phenomena, making it a global problem. This fact is enhanced when large population groups coexist, as happens in crowded refugee camps or in war and conflict areas, before the populations dissemination to their host countries [22]. Leishmaniasis can manifest in humans mainly in three different clinical conditions: visceral, the most aggressive and mortal form; cutaneous, leading to nodules and ulcers in the skin; and mucocutaneous, which causes permanent lesions in the mucosa (mouth, nose or genital) [23,24]. In all cases, the transmission vectors are dipteral insects of the genera *Lutzomyia* in the New World and *Phlebotomus* in the Old World (Table 1) [25]. Leishmaniasis is considered to be a neglected disease because of the low investment in the search for new, efficient drugs. Glucantime is the only drug available to fight these diseases nowadays [26,27], since the use of Pentostam is deprecated [28]. On the other hand, Chagas disease (or American trypanosomiasis) [29] is a chronic neglected illness caused by a *Trypanosoma cruzi* parasite, and affecting more than 10 million people worldwide. Its main incidence region comprises poor South and Central America areas, but the infection is present in many other continents through migration patterns and blood transfusions from latent donors. The transmission mechanism runs through contact with urine and faeces of infected blood-sucking triatomine bugs [30,31]. The most commonly used commercial drugs to fight against these infections are nifurtimox and benznidazole, two nitro heterocyclic derivatives with contrasted trypanozidal effects [32,33,34]. In the past three decades, many biochemical studies have pointed to different possible targets to stop the infections, like the inhibition of ADP phosphorylation or the beta-oxidation of fatty acids [35,36]. Despite the obvious advantages of these commercial drugs and the fact that they are still the first-line antiparasitic treatments, they exhibit several limitations, like serious side effects [37]. Thus, the development of novel and more efficient therapies to fight Chagas disease, minimizing its impact in society, is also an urgent need [38,39,40].

The molecular structures of antimonial drugs like meglumine antimoniate (Glucantime), as well as their modes of action and metabolic natures, are still being explored [41,42,43]. Alternatives with non-antimonial antiparasitic prodrugs such as anfotericine B are also being used as leishmaniasis treatments [44,45], but their prices restrict their availability and implementation in underdeveloped countries, which are the most affected areas. Moreover, these non-antimonial prodrugs present adverse side effects, especially kidney failure, in addition to the beginning of resistance, as reported last year [46]. The antimonial drugs also present serious side effects, like allergic dermopathy, peripheral polyneuropathy or vomiting [47], the need for daily parenteral administration, and the development of drug resistance [48,49]. Many research groups are making advances in this field by using transition-metal complexes to combat parasitic diseases [50,51], mainly with ruthenium compounds [52,53,54,55,56]. Antiparasitic evaluation studies and intra- and extracellular assays have been carried out by our research group using 1,2,4-triazolo[1,5-a]pyrimidine derivatives and their metal complexes, studies which have shown great results against tropical diseases currently without effective treatments, and particularly against leishmaniasis and Chagas disease [57,58,59,60,61]. Many other authors have also pointed to a metal-complexes strategy as a promising alternative for fighting tropical parasitic diseases caused by members of the *Trypanosomatidae* family, such as leishmaniasis, as well as other tropical illnesses like Chagas disease or malaria. A synergetic effect is observed in antiparasitic efficacy when the triazolopyrimidine derivatives are combined with a variety of metal ions in coordination complexes [62,63]. This renders triazolopyrimidine metal complexes potential chemotherapeutic agents for curing diseases related to parasites. The interest in this topic has led to some reviews being published in the last decade [64,65,66].

The use of and interest in nanomaterials in biomedicine is increasing every year due to their characteristic mechanical, optical and electrical properties [67,68], especially as carriers for drug delivery [69]. Focusing on that, nanoparticles are some of the most widely used transporters. The possibility of size and porosity control in their synthesis, as well as their versatility of functionalization make them one of the most useful types of carriers to distribute drugs in an efficient way [70,71]. Their nature can range from polymer- [72] or protein-based [73] to metallic gold [74], iron [75], or copper nanoparticles [76]. Mesoporous silica nanoparticles are one of the most-used nanosystems in biomedicine for drug delivery [77,78,79], despite the fact that their use in parasitology has just bloomed in the last five years [80,81,82].

In the present work, luminescence properties, solution stability and biological assays of four 7-oxo-5-phenyl-1,2,4-triazolo[1,5-a]pyrimidine (**HftpO**) first-transition complexes with the general formula [M(ftpO)_2_(H_2_O)_4_] with M = Cu (**1**), Co (**2**), Ni (**3**), and Zn (**4**) are discussed for the first time. The antiparasitic activity of **HftpO** complexes was evaluated against an Arequipa strain of *T. cruzi* and five *Leishmania* spp. species (the European type *L. infantum*; the South American parasites *L. braziliensis* present in Brazil, *L. peruviana* indigenous to Peru, and *L. mexicana*, which acts mainly in the Yucatán Peninsula; and the mostly-reported-in-the-Indian-subcontinent *L. donovani*), as well as evaluated for toxicity against macrophage J774.2 and Vero host cells. The results reveal a better selectivity index in most cases, compared with the isolated ligand and the commercial drugs Glucantime and benznidazole. Thus, a proof-of-concept of drug release for these kinds of complexes using silica nanoparticles as carriers was performed using the copper one as model, confirming that the complex antiparasitic capacity continues to be active against different *Leishmania* spp. species.

## 2. Results

### 2.1. Synthesis and Characterization of Triazolopyrimidine Complexes

The four metal complexes of 7-oxo-5-phenyl-1,2,4-triazolo[1,5-a]pyrimidine (**HftpO**) were synthesized following the method previously reported by our group [21]. The general procedure can be found in the *Materials and Methods* section of this work.

#### 2.1.1. Crystal Structures of [M(ftpO)_2_(H_2_O)_4_] (**1**–**4**)

**Figure 1:**
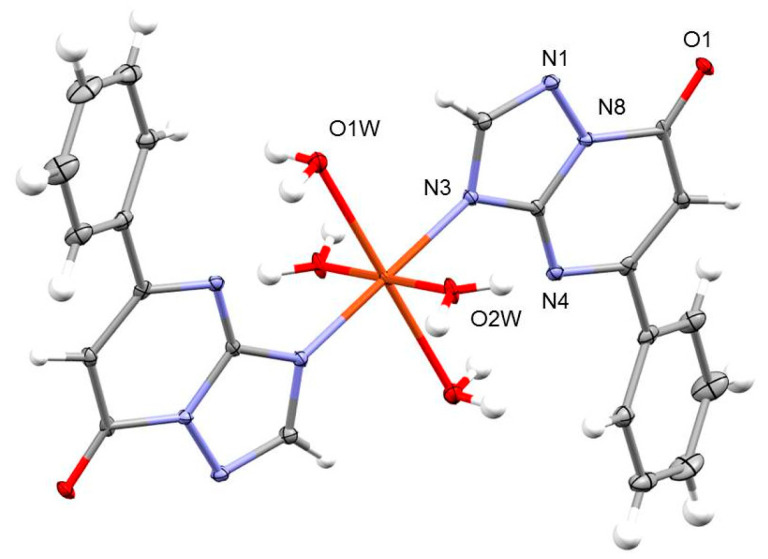
Perspective view of **[Cu**(**ftpO**)**_2_**(**H_2_O**)**_4_]** (**1**). Thermal ellipsoids are drawn at the 50% probability level. Reproduced with permission from [21], Elsevier, 2017.

Compound **1** crystallizes in the monoclinic *P*2_1_/c space group. A perspective view of this complex is shown in Figure 1. The structure consists of one mononuclear [Cu(ftpO)_2_(H_2_O)_4_] coordination complex, in which each Cu (II) ion has an octahedral CuN_2_O_4_ stereochemistry. This geometry is constituted by four coordination water molecules and two nitrogen atoms belonging to two different anionic **ftpO^−^** ligands. A strong hydrogen-bond interaction between O1W and N4 is also present, leading both coordinated **ftpO^−^** moieties up to an almost coplanar position. The crystallographic data of compound **1** were previously reported by our group [21], and the whole dataset can be obtained free of charge from The Cambridge Crystallographic Data Centre via “www.ccdc.cam.ac.uk/data_request/cif accessed on 25 September 2023” linked to identification number 1536813. X-ray powder diffraction (XRD) data confirm the isostructural nature of the four complexes, assigning the general formula [M(ftpO)_2_(H_2_O)_4_] to the whole series.

#### 2.1.2. Spectroscopic Properties

**Figure 2:**
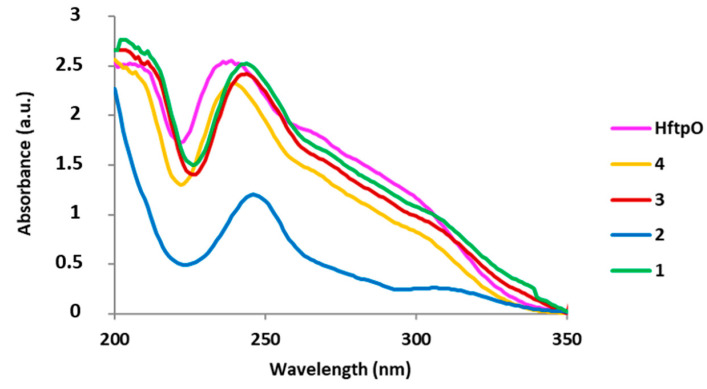
Comparison between UV spectra of **HftpO** ligand and [M(ftpO)_2_(H_2_O)_4_] [M = Cu (**1**), Co (**2**), Ni (**3**), and Zn (**4**)] complexes in ethanol.

In order to complete the spectroscopic studies of these complexes, stability over time was measured by UV using 10^−3^ M ethanol absolute solutions of the four complexes. The spectra were recorded across 3 days at different moments, maintaining the curves in the same shape after 72 h in solution (Figure 2). Absorption maximums for each complex were distorted from the **HftpO** maximum, indicating that complex integrity remains unaltered in the ethanol solution (Table 2).

**Figure 3:**
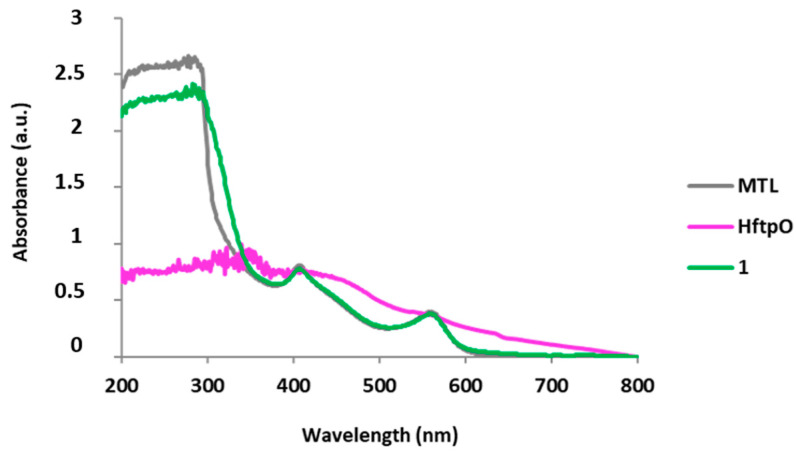
Comparison between UV spectra of MTL (grey), free **HftpO** (pink) and complex **1** (green). Only one complex spectrum is included in this comparison for simplicity, since the shape remains the same for the 4 metal complexes synthesized.

UV behaviour was also studied for the samples in vitro, in trypanosomal liquid medium (MTL) solution after two days of heating at 37 °C, via ultrasounds in sealed Eppendorf tubes. As can be noticed in Figure 3, the curves corresponding to MTL without sample, the sample containing **HftpO** and the sample containing the [Cu(ftpO)_2_(H_2_O)_4_] complex exhibit different shapes. The four metal compounds have identical spectrum shapes, a result which supports the isostructural nature of the samples (a description can be found in the Supplementary Material, Figures S1–S3). The **HftpO** ligand presents a maximum of around 280 cm^−1^ in MTL, while for the metal compounds, this value shifts to 300 cm^−1^, supporting the stability-in-solution of the complexes in a parasite culture medium.

Complementary IR spectra information, as well as a complete thermoanalytical study of the complexes **1**–**4**, can be found in Mendez-Arriaga et al. [21].

#### 2.1.3. Luminescent Properties

**Figure 4:**
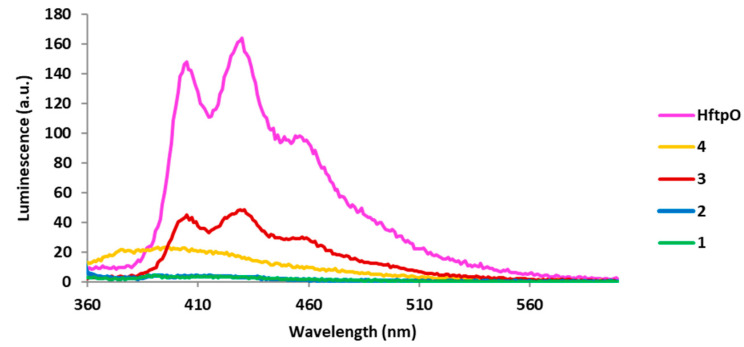
Comparison between the luminescent emission spectrum of free **HftpO** derivative (red) and its complexes **1**,**2** (yellow and blue, coincident), **3** (purple) and **4** (green).

The luminescent emission spectra of free **HftpO** and its metal complexes were recorded in ethanol solution (1 × 10^−3^ M) with an excitation wavelength of 348 nm at room temperature. The maximum UV-absorption wavelength previously obtained for each compound was tested, but no emission signals were obtained. These emission spectra are depicted in Figure 4. Free-ligand **HftpO** exhibits a strong luminescent emission between 400–440 nm, in contrast to the partial luminescence-quenching phenomena observed in the nickel complex (**3**). In the cases of complexes **1**,**2**, with Cu(II) and Co(II) centres, the luminescence signal fully disappears. The zinc complex (**4**) exhibits a different emission pattern in comparison with the cobalt one and **HftpO**, at 348 nm. The decreases in emission intensity were in the order of Co(II) = Cu(II) > Zn(II) > Ni(II). The decrease of the luminescence signal is a rather common phenomenon attributed to the interaction between metal ions and strongly luminescent ligands, such as Schiff bases or aromatic heterocycles, during the complexation process [83].

### 2.2. Synthesis and Characterization of Functionalized Silica Nanoparticles

**Scheme 2:**
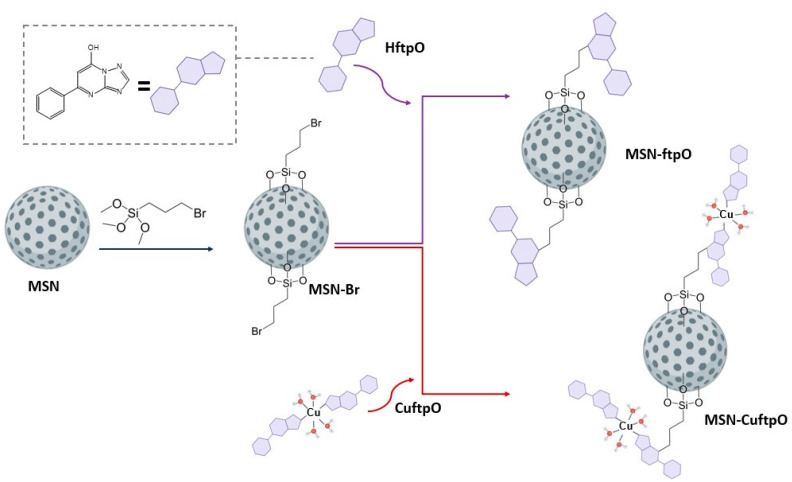
Schematic procedure for the immobilization of **HftpO** and model **CuftpO** complex **1** onto the MSN surface.

The synthesis of MCM-41 silica nanoparticles (**MSN**) was carried out following the procedure reported by Zhao et al. [84], with slight modifications. To enhance the coordination capacity of the proposed nanosystems, **MSN** nanoparticles were covered with bromide groups from bromopropyl triethoxysilane ligand to obtain **MSN-Br** systems. Finally, as a proof of concept, **HftpO** ligand and complex **1** were selected for immobilisation on the MSN surface for further release in order to study kinetics and evaluate their efficacy as leishmanicidal agents. A standard procedure of stir-and-heat in toluene in a Schlenk tube with a nitrogen atmosphere were performed to obtain **MSN-ftpO** and **MSN-CuftpO** nanosystems (Scheme 2). The functionalization of silica nanoparticles was performed following the methodology previously reported by our group [70], which can be found in the Section 4.

#### 2.2.1. Transmission Electron Microscopy (TEM)

**Figure 5:**
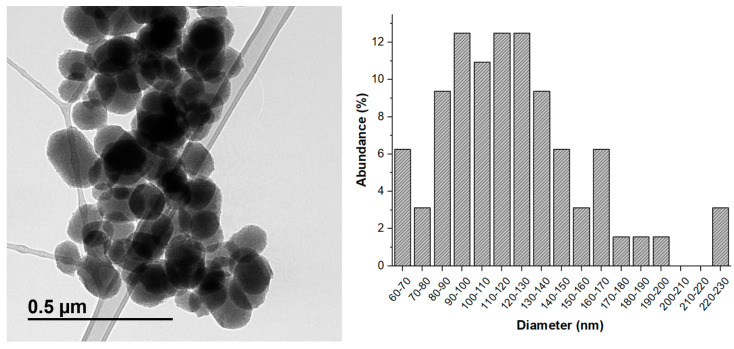
MSN TEM micrography (**left**) and mean diameter size-distribution of MSN (**right**).

To evaluate the nanoparticles’ appearance, mesoporous silica nanoparticle (**MSN**) starting material was characterized by TEM. As shown in Figure 5, **MSN** presents a semi-spherical morphology with a wide size distribution, with a diameter prevalence between 110 and 130 nm. A few, larger, 220–230 nm nanoparticles can be noticed in the histogram, but these data can be attributed to the fusion of small silica nanoparticles. Well-defined pores in a hexagonal arrangement can be observed, a common characteristic of MCM-41 MSN.

#### 2.2.2. Nitrogen Adsorption/Desorption Isotherms

**Figure 6:**
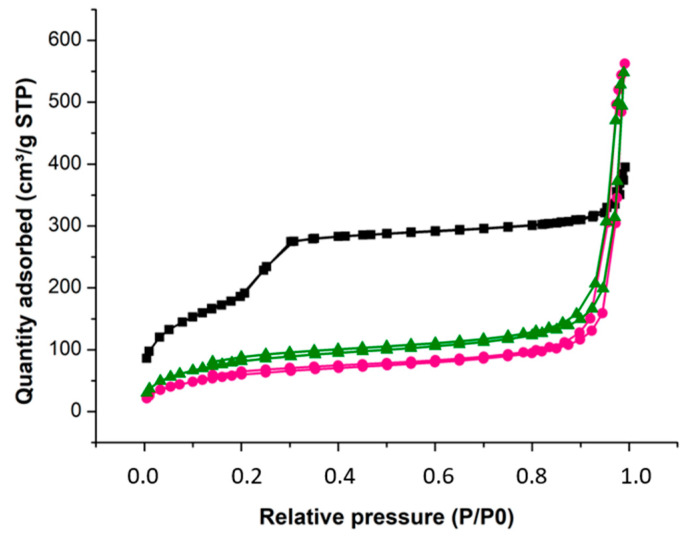
N_2_ adsorption/desorption isotherms of **MSN** (black) **MSN-ftpO** (pink) and **MSN-CuftpO** (green).

The porous properties of the starting material (**MSN**) and both functionalized carriers (**MSN-ftpO** and **MSN-CuftpO**) were evaluated with nitrogen adsorption/desorption isotherms at 77 K, as shown in Figure 6. Experimental results show a decrease in the surface area of the modified **MSN** compared to the starting material, as expected. **MSN** exhibits the classical type IV isotherm (characteristic of mesoporous silica supports), while both functionalized nanosystems exhibit the characteristic appearance of materials with filled pores [85,86]. Data-fitting of the nitrogen adsorption/desorption results to Brunauer–Emmett–Teller (BET) isotherms permitted the determination of the most relevant surface parameters (Table 3). Raw **MSN** presented a BET surface of 682 m^2^g^−1^, a pore volume of 0.52 cm^3^g^−1^ and a 3.2 nm pore diameter. The functionalization of **MSN** surface with Br and the subsequent anchoring of the **HftpO** ligand and complex **1** resulted in a decrease of the three parameters in both studied nanomaterials. Surface area and total pore volume presented slightly higher values in **MSN-CuftpO** due to the steric hindrance of the larger size of the metallic complex occupying the available pores of the silica.

### 2.3. Release Studies

**Figure 7:**
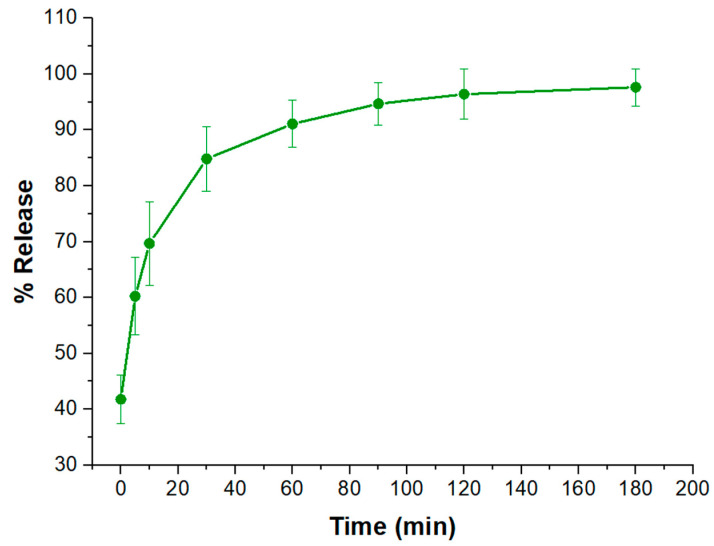
Kinetic release profile of the copper complex (**1**) from **MSN-CuftpO.** Data are shown only up to 3 h for simplicity.

The release behaviour of copper complex (**1**) was studied by a specific assay of **MSN-CuftpO**. Thus, three different samples containing 0,4 mg copper complex nanomaterial were suspended in 4 mL of PBS buffer (0.1 mg/mL, pH 7.4). This buffer was chosen as a model to mimic the cell culture media that was subsequently used, which was coherent with human pH, and to avoid contamination using MTL. After an initial sonication, the sample was incubated at 37 °C and centrifuged at specific time intervals (Figure 7), in order to analyse the absorption via UV spectroscopy at the maximum absorbance wavelength of complex **1**. The experiment was conducted for 3 days, but the stabilization was reached at 3 h, verifying the complete release of the loaded compound.

### 2.4. In Vitro Antiparasitic Activity and Toxicity

#### 2.4.1. In Vitro Efficacy of HftpO and Its Complexes (**1**–**4**)

**Figure 8:**
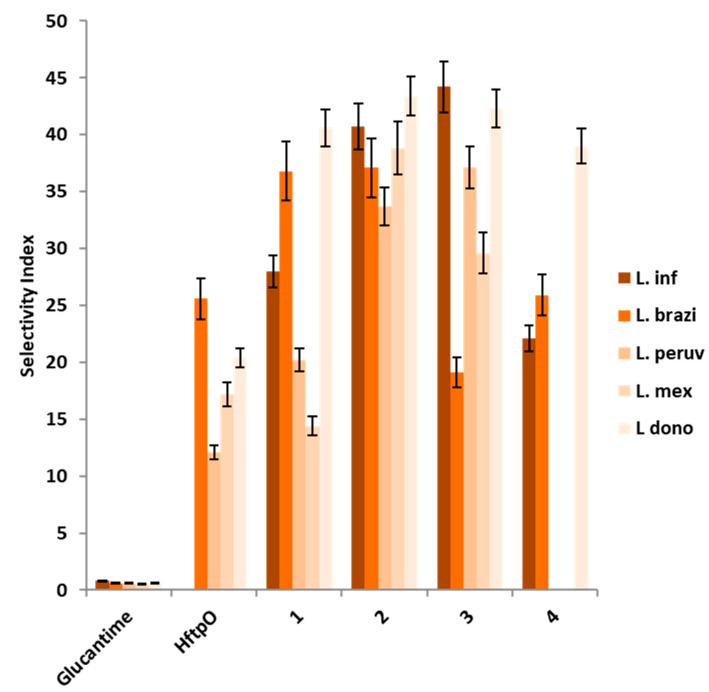
Comparative SI values for *Leishmania* spp. strains between Glucantime, free **HftpO** ligand, and complexes **1**–**4**.

**Figure 9:**
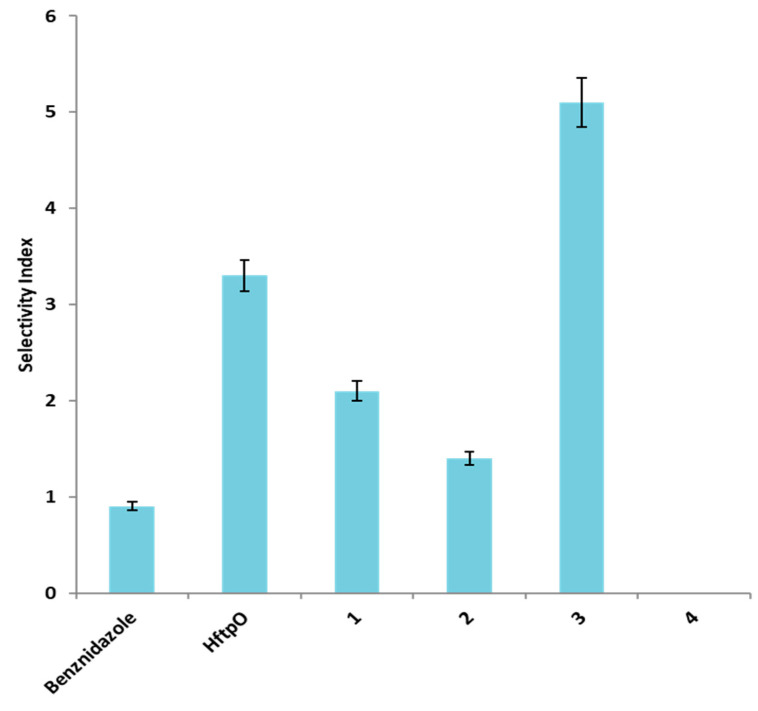
Comparative SI values for *Trypanosoma cruzi* between benznidazole, free **HftpO** ligand, and complexes **1**–**4**.

Taking into account the synergic properties of the metallic complexes with triazolopyrimidine ligands, as previously described by several authors [64,65,66], and given that currently used treatments against leishmaniasis usually cause serious problems, we decided to check the antiproliferative activity and cytotoxicity of the four mentioned complexes against five different species of *Leishmania* spp. (*L. infantum*, *L. braziliensis*, *L. peruviana*, *L. mexicana* and *L. donovani*) and *Trypanosoma cruzi*. The results were compared with commercial drugs Glucantime and benznidazole, isolated **HftpO** derivative, and inorganic salts employed in the synthesis (Table 4 and Table 5, Figure 8 and Figure 9) in order to study the potential efficacy improvement provoked by the metal coordination. According to the promastigote growth inhibition, no significant effect was noticed for the inorganic salts, as expected, so the data are not included in the tables.

#### 2.4.2. In Vitro Screening of MSN-ftpO and MSN-CuftpO

**Figure 10:**
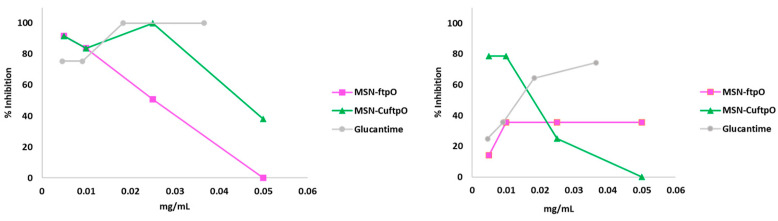
Comparative SI values for *Leishmania infantum* (**left**) and *Leishmania braziliensis* (**right**) between Glucantime (grey), **MSN-ftpO** (pink) and **MSN-CuftpO** (green).

Cytotoxicity assays and promastigote screenings were performed for the silica nanoparticles and their derived nanomaterials to check their capability to inhibit *leishmania* spp. growth. *L. infantum* and *braziliensis* were selected as parasite models of two different clinical behaviour infections, and were incubated with **HftpO** and copper complex **1** silica nanomaterials. Compound **1** was selected due to its promising behaviour against both studied *Leishmania* spp. species and a significant efficacy against Chagas disease. Table 6 exhibits the results of these biological assays. The inhibition tendency of the studied materials is represented in Figure 10.

## 3. Discussion

The **HftpO** ligand showed a significant effect on the extracellular parasite forms, but its IC_50_ was higher than those of the reference drugs. The metallic complexes presented similar acceptable values for IC_50_ in the promotion-of-cytotoxicity assays, but they still did not exceed the performance of commercial drugs in some cases, like that of *L. braziliensis*. Nevertheless, for the **HftpO** ligand as well as its metal complex, the cytotoxicity results were better than those of the reference drugs. Comparing Glucantime and **HftpO**, the concentration needed to consider the triazolopyrimidine toxic for the host cells was more than 60-fold higher than that of the commercial leishmanicidal drug (Table 4, IC_50_ against J774.2 macrophages column). This improvement of the toxicity effect was incrementally achieved in the complexes due to the synergetic effect derived from the coordination to a metal ion centre. For all of the metallic compounds, the cytotoxicity IC_50_ values for macrophages were extraordinarily high. On the other hand, the Vero cells assay resulted in very strong cytotoxicity values which surpass the Benznidazole ones, but the free **HftpO** still exhibits the lowest toxic activity for Vero cells. The selectivity index (SI) is obtained by the combination of promastigote inhibition growth and cytotoxicity in host cells and is used to evaluate the efficacy of a prodrug [87]. The mentioned coefficient illustrates the great efficacy of these **HftpO** complexes as anti-leishmania agents, improving upon values in comparison to the commercial drug and free ligand in all cases. Figure 8 and Figure 9 depict and compare the SI values for all the compounds assayed against different *Leishmania* spp. species and *Trypanosoma cruzi*, respectively.

As shown in Figure 8, nickel and cobalt complexes (**2** and **3**) exhibit the best SI values of the series, being more than 50-fold better than Glucantime values for every single *Leishmania* spp.-assayed species, with only the exception of **3** for *L. braziliensis.* Nevertheless, focusing on the *L. braziliensis* inhibition, the Cu(II) coordination complex (**1**) possesses the best ratio of efficacy for fighting this illness. The only compound with an apparently worse activity is **4**, which is in good agreement with the previously reported data for zinc triazolopyrimidine complexes [65]. Nevertheless, if we compare the published data of the only two zinc 1,2,4-triazolo[1,5-a]pyrimidine complexes already assayed against *Leishmania* spp. species, [Zn(dmtp)_2_(H_2_O)_4_](ClO_4_)_2_·2dmtp·2H_2_O [11] and [Zn_2_(7atp)_4_(µ-bypym)(H_2_O)_4_](ClO_4_)·2(7atp) [88], this new zinc compound improves the SI result for *L. infantum*, *L. braziliensis* and *L. donovani*, thus becoming the best zinc triazolopyrimidine compound with antiparasitic effectiveness yet reported, as far as we know. The *T. cruzi* SI values show that complex **3** is the best antiparasitic prodrug, followed by the free ligand. Only the zinc compound under discussion in this work turns out to be an exception, with no significant antiproliferative efficacy shown against *T. cruzi*, *L. peruviana* or *L. mexicana.* This phenomenon can be attributed to the general tendency towards a low solubility in Zn complexes in comparison with other transition-metal compounds [89,90], which could prevent a correct interaction in solution with the parasite species studied.

In general, **HftpO** coordination compounds display better SI coefficients against *Leishmania* spp. strains, not only compared to Glucantime, but also surpassing the free triazolopyrimidine derivative. In the case of Chagas disease, only the nickel complex improves upon the ligand results, but the commercial reference is always surpassed by Cu, Co and Ni. This fact supports the theory of a synergetic effect caused by the coordinated combination between metal centres and organic ligands, improving the effectiveness against parasite infections by associating it with a decrease in the toxicity levels tolerated by the host cells.

The results for the antiparasitic activity of the nanomaterials evaluated indicate a great efficacy of the copper complex, with higher values of SI in comparison with free Glucantime, while keeping a similar efficacy range, despite the fact that silica nanoparticles were modified with only 20% of the ligand or metal complex, relative to the weight percentage. The release profile shows a complete liberation of the drug inside of two hours, confirming the complete administration of the complex during the screening experiment. A prevailing trend at low concentrations for both proposed systems can be noticed by looking at the inhibition graphics (Figure 10), which are especially pronounced for *L. braziliensis*. This behaviour can be attributed to an agglomeration tendency for silica nanomaterials in the MTL medium in the experiment wells in scalation concentrations.

## 4. Materials and Methods

### 4.1. General

All reagents were purchased from commercial sources and used as received without further purification. The synthesis of HftpO ligand and its metal complexes followed the methodology described in Méndez-Arriaga et al. [21], which is briefly described below.

### 4.2. Synthesis of HftpO and [M(ftpO)_2_(H_2_O)_4_] where M(II) = Cu (***1***), Co (***2***), Ni (***3***) and Zn (***4***)

Following the procedure previously reported by our research group [21], a mixture containing ethyl benzoylacetate (0.02 mol) and 3-amino-1,2,4-triazole (0.02 mol) was refluxed in 10 mL of acetic acid for 12 h. A white solid appeared when cooling, which was washed with abundant water and CH_2_Cl_2_ to remove all the acid excess. Then, the white solid was dried in a vacuum desiccator. For the synthesis of the metal complexes, a hot solution of HftpO (1 mmol) in 20 mL of ethanol was added to another warm solution of 5 mL of ethanol containing 1 mmol of the respective nitrate salt (1:1). First precipitate, which was formed immediately, was removed from the solution. After one week at room temperature, a microcrystalline product was obtained.

### 4.3. Synthesis of Mesoporous Silica Nanoparticles

The synthesis of MCM-41 silica nanoparticles (MSN) was carried out following the procedure reported by Zhao et al. [84], with slight modifications. A total of 3.5 mL of a 2 M aqueous sodium hydroxide solution was added to an aqueous solution of CTAB (1.0 g, 2.74 mmol in 480 mL of Milli-Q water). After that, the silica precursor TEOS (5 mL, 22.4 mmol) was added dropwise under vigorous stirring, allowing the mixture to react for 2 h at 80 °C. Once a white precipitate appeared, the product was isolated by filtration, washed with abundant Milli-Q water and methanol (2 × 20 mL) and dried for 24 h at 80 °C on a stove. Finally, the MSN were calcined at 550 °C for 24 h with an increasing temperature ramp of 1 °C/min.

### 4.4. Functionalization of Mesoporous Silica Nanoparticles with (3-Bromopropyl)triethoxysilane (MSN-Br)

**MSN** nanoparticles were functionalized with bromide groups. This process was performed following the procedures reported previously by Bollu et al. [91]. A quantity of 500 mg of MSN in a Schlenk tube was dehydrated at 90 °C under vacuum overnight. The activated silica was dispersed in dry toluene (40 mL), adding 525 µL of bromopropyl triethoxysilane ligand (BrP) (100% *w*/*w* BrP/SiO_2_) to the solution. The mixture was heated to 110 °C and stirred for two days. After that, the dispersion was centrifuged and the solid was washed with toluene (×2) and with diethyl ether (×1). The obtained white solid, MSN-Br, was dried overnight at 70 °C on a stove.

### 4.5. Ligand and Complex Coordination (MSN-ftpO and MSN-CuftpO)

**HftpO** ligand and copper complex 1 were incorporated into the silica nanomaterial MSN by coordination through HBr liberation. Initially, 100 mg of MSN-Br was activated at 90 °C and under vacuum in a Schlenk tube overnight. After that, 20 mL of dry toluene and 20 mg of ligand or complex, respectively, were added and heated for 48 h at 110 °C. Finally, the suspension was centrifuged, and the isolated solid was washed twice with toluene and once with diethylether. Finally, the solids were dried at room temperature, obtaining a 20% in weight functionalization.

### 4.6. Physical Measurements

Luminescence and UV spectra for ethanol solutions were recorded with Varian Cary Eclipse equipment at University of Granada. Adsorption−desorption isotherms of nitrogen were measured using a Micromeritics ASAP 2020 at University Rey Juan Carlos. Surface areas and pore size were calculated using Brunauer–Emmett–Teller (BET) and Barret–Joyner–Halenda (BJH) methods, respectively. Transmission electron microscopy (TEM) images were obtained with a JEOL JEM 1010 at a 100 kV operating voltage. The samples were dispersed using ethanol solvent, followed by an ultrasonic bath for 30 min before being spread onto a TEM copper grid (300 mesh) covered with a holey carbon film. The grid was then air-dried at room temperature. The images were acquired with the equipment’s software and managed with ImageJ, version 1.53t [92].

### 4.7. Parasite Strain Culture

Extracellular forms of the studied parasites (known as promastigotes in Leishmania and epimastigotes in *T. cruzi*) were cultured in a liquid culture medium following the methodology described in González et al. [93]. Studied strains were obtained from: *T. cruzi* (MHOM/Pe/2011/Arequipa, DTU V), *L. peruviana* (MHOM/PE/84/LC26), *L. mexicana* (MHOM/BZ/82/Bel21), *L. braziliensis* (MHOM/BR/1975/M2904), *L. donovani* (MHOM/PE/84/LC26) and *L. infantum* (MCAN/ES/2001/UCM-10). All of these strains were transferred to our research group by Laboratorio de Leishmaniosis y Chagas, Instituto Nacional de Salud, Lima (Peru).

### 4.8. In Vitro Activity Assays

The screening of extracellular forms of parasites was carried out using 24-well plates with MTL medium and 5 × 10^4^ parasites per well. HftpO complexes present serious solubility problems due to stacking interactions between the phenyl groups of the ligand, so the dilution was not possible in water or biologically compatible mixtures of ethanol, DMF or DMSO. The samples were finally suspended in culture medium after two days of moderate heating at 37 °C and ultrasounds in sealed Eppendorf tubes. Afterwards, they were tested at 1, 10, 25 and 50 μM, leaving some wells without drugs as control, and were incubated at 28 °C during 72 h before the parasite final count by haemocytometric Neubauer chamber.

### 4.9. Cell Culture and Cytotoxicity Test

Macrophages were used as cellular models to simulate host cells, due to the fact that those are the usual cells Leishmania parasites infect. J774.2 macrophages from the European Collection of Cell Culture (ECACC, number 85011428) were cultured in RPMI medium supplemented with 10% inactivated calf serum and maintained at 37 °C with a 5% CO_2_ atmosphere. Vero cells (EACC, number 84113001), chosen with the same purpose as that of the *T. cruzi* studies, underwent the same culture conditions. All the mammal cells used in the cytotoxicity test were purchased from Centro de Instrumentación Científica, University of Granada (Spain) (https://cic.ugr.es/, accessed on 25 September 2023).

Cytotoxicity tests were performed in 96-well microplates using compound concentrations of 50, 100, 200 and 400 μM. Resazurin blue was added to each well in order to measure colour intensity using an ELISA reader.

The half maximal inhibitory concentration (IC_50_) is a quantitative measure of the potential of a compound to inhibit a specific biological process by 50%, and is primarily defined through in vitro test models [93]. Selectivity index (SI) can be defined as the ratio of the toxic concentration of a sample against its effective bioactive concentration [94,95].

## 5. Conclusions

Four triazolopyrimidine metal complexes have been characterized by spectroscopic techniques, verifying the coordination of the triazolopyrimidine ligand to the metal centres, and their biological activity was studied. The tested coordination complexes improved upon the previously reported leishmanicidal effect of commercial drugs and free **HftpO** ligand in most of the species analysed, showing much less toxicity against hosting macrophage and Vero cells, despite the fact that they present lower antipromastigote activity, upholding the proposed synergetic effect of the combination of a metallic centre with tryazolopyrimidine derivatives. Cobalt complex **2** seems to be the most versatile compound for different *Leishmania* spp. species, together with copper complex **1**, due to the homogeneity of results between the two strains. Nanoparticles with an **HftpO** coordinated copper complex have shown an interesting profile of high efficacy at very low concentrations and a similar profile to those of commercial drugs, even with only 20% of the active compound in their surfaces. The assayed materials have turned out to be great candidates for further biological assays in the search for side-effect-free drugs, as well as multifunctional materials which can incorporate key molecules to facilitate the interaction between the drug candidates and biological targets.

## Figures and Tables

**Table 1:** *Leishmania* spp. species used in this work, with their geographical locations, reservoir hosts and vectors.

Leishmania Species	Infection	Geographical Location	Reservoir	Vector
*L. infantum*	VL, CL, ML	New World for VL Old World for CL and ML	Rodents, dogs, foxes, jackals	*L. longipalpis* *P. pcrniciosufi*, *P. arias*
*L. braziliensis*	CL, MCL	New World	Forest rodents, anteaters	*Lutzomyia* spp.
*L. peruviana*	MCL	New World	Dogs	*L. verrucarum*, *L. pvmenis*
*L. mexicana*	CL, DCL, MCL	New World	Forest rodents	*L. olmeca*
*L. donovani*	VL, PKDL	Old World	Humans, rodents	*P. argentipes*, *P. orinntalis*, *P. martini*

VL: Visceral leishmaniasis; CL: Cutaneous leishmaniasis; ML: Mucosal leishmaniasis; MCL: Mucocutaneous leishmanisis; DCL: Diffuse cutaneous leishmaniasis; PKDL: Post-kala azar dermal leishmaniasis. Data in Table 1 were obtained from WHO databases and Oryan et al. [25], Shiraz University, 2015.

**Table 2:** Maximum absorption peaks for **HftpO** and complexes **1**–**4**.

Compound	Wavelength (nm)
**HftpO**	238
**1**	245
**2**	244
**3**	246
**4**	240

**Table 3:** Surface parameters of **MSN**, **MSN-ftpO** and **MSN-CuftpO** obtained by N_2_ adsorption/desorption analysis.

Material	S_BET_ (m^2^g^−1^)	Total Pore Volume (cm^3^g^−1^)	Pore Diameter (nm)
**MSN**	682	0.52	3.2
**MSN-ftpO**	227	0.47	<2.0
**MSN-CuftpO**	305	0.49	<2.0

**Table 4:** In vitro activity of complexes (**1**–**4**) against promastigote forms of *Leishmania* spp. and J774.2 macrophages after 72 h of incubation at 37 °C.

Compound	IC_50_ (µM) ^a^ ± SD						SI ^b^				
	*L. inf.*	*L. brazi.*	*L. peruv*	*L. mex.*	*L. dono.*	J774.2 macro.	*L. inf.*	*L. brazi.*	*L. peruv.*	*L. mex*.	*L. dono.*
Glucantime	18.0 ± 3.1	25.6 ± 1.7	25.0 ± 2.0	31.0 ± 2.3	26.6 ± 5.4	15.2 ± 1.0	0.8	0.6	0.6	0.5	0.6
**HftpO**	>200	37.5 ± 2.9	79.0 ± 6.7	116.6 ± 10.2	46.9 ± 3.8	958.5 ± 74.2	-	25.6 (42)	12.1 (20)	17.2 (34)	20.4 (34)
**1**	71.5 ± 5.8	54.4 ± 3.6	99.2 ± 8.7	139.3 ± 11.6	49.3 ± 3.9	1827.0 ± 138.6	25.6 (32)	33.6 (56)	18.4 (31)	13.1 (26)	37.1 (62)
**2**	49.1 ± 4.4	53.9 ± 4.1	59.3 ± 6.6	51.6 ± 4.5	46.1 ± 3.3	1200.8 ± 111.4	24.5 (31)	22.3 (37)	20.2 (34)	23.3 (47)	26.0 (43)
**3**	45.2 ± 3.8	104.8 ± 7.4	53.9 ± 5.1	67.6 ± 5.4	47.2 ± 3.7	2438.6 ± 156.3	53.9 (67)	23.3 (39)	45.2 (57)	36.1 (72)	51.7 (86)
**4**	90.3 ± 6.1	77.3 ± 4.0	>200	>200	51.3 ± 3.2	1026.9 ± 89.4	11.4 (14)	13.3 (22)	-	-	20.0 (33)

Three different determinations were measured in each case to elaborate the table. ^a^ IC_50_ = Concentration required to obtain a 50% inhibition in parasite or cell population, calculated with a linear regression analysis from the values employed (1, 10, 25 and 100 µM for promastigote forms of *Leishmania* spp. and 50, 100, 200 and 400 µM for host cells). ^b^ Selectivity index = IC_50_ against J774.2 macrophages/IC_50_ parasite (promastigote forms). In brackets: number of times that ligand SI surpassed the reference drug SI.

**Table 5:** In vitro activity of complexes (**1**–**4**) against epimastigote forms of *T. cruzi* and Vero cells after 72 h of incubation at 37 °C.

Compound	IC_50_ (µM) ^a^ ± SD		SI ^b^
	*T. cruzi*	Vero Cell	*T. cruzi*
Benznidazole	15.5 ± 1.4	13.6 ± 0.9	0.9
**HftpO**	173.0 ± 16.7	573.0 ± 43.8	3.3 (4)
**1**	153.1 ± 13.2	322.8 ± 26.8	2.1 (2)
**2**	112.5 ± 9.8	156.9 ± 11.4	1.4 (1)
**3**	73.5 ± 6.8	375.8 ± 32.7	5.1 (6)
**4**	>200	-	-

Three different determinations were measured in each case to elaborate the table. ^a^ IC_50_ = Concentration required to obtain a 50% inhibition in parasite or cell population, calculated with a linear regression analysis from the values employed (1, 10, 25 and 100 µM epimastigote forms of *T. cruzi* and 50, 100, 200 and 400 µM for host cells). ^b^ Selectivity index = IC_50_ against Vero cells/IC_50_ parasite (epimastigote forms). In brackets: number of times that ligand SI surpassed the reference drug SI.

**Table 6:** In vitro activity of nanomaterials against promastigote forms of *Leishmania* spp. and J774.2 macrophages after 72 h of incubation at 37 °C.

Compound	IC_50_ (µg/mL) ^a^ ± SD			SI ^b^	
	*L. inf.*	*L. brazi.*	J774.2 macro.	*L. inf.*	*L. brazi.*
Glucantime	14.0 ± 1.4	6.0 ± 0.7	17.2 ± 1.0	1.2	2.9
**MSN**	-	-	>1000	-	-
**MSN-Br**	-	-	54.6 ± 4.9	-	-
**MSN-ftpO**	25.0 ± 3.1	33.0 ± 3.8	55.4 ± 5.1	2.1 (2)	1.7 (0)
**MSN-CuftpO**	16.0 ± 0.8	19.0 ± 1.0	162 ± 18.6	8.7 (7)	8.5 (3)

Three different determinations were measured in each case to elaborate the table. ^a^ IC_50_ = Concentration required to obtain a 50% inhibition in parasite or cell population, calculated with a linear regression analysis from the values employed (1, 10, 25 and 100 µM for Glucantime and 0.025, 0.01, 0.005 mg/mL for nanomaterials). ^b^ Selectivity index = IC_50_ against J774.2 macrophages/IC_50_ parasite (promastigote forms). In brackets: number of times that ligand SI surpassed the reference drug SI.

## Data Availability

Data is contained within the article and supplementary material.

## Supplementary Materials

The following supporting information can be downloaded at: https://www.mdpi.com/article/10.3390/ph16101380/s1, Figure S1. UV spectra complex 2 in MTL medium; Figure S2. UV spectra complex 3 in MTL medium; Figure S3. UV spectra complex 4 in MTL medium.
