# Supplementary material for: First-Row Transition 7-Oxo-5-phenyl-1,2,4-triazolo[1,5-a]pyrimidine Metal Complexes: Antiparasitic Activity and Release Studies
Source: Pharmaceuticals (Basel). 2023 Sep 28;16(10):1380. doi: 10.3390/ph16101380 (PMC10610057; doi:10.3390/ph16101380)
Supplement: Supplementary file 1 [file pharmaceuticals-16-01380-s001.zip › pharmaceuticals-2612260-supplementary.pdf]

First-Row Transition 7-Oxo-5-phenyl-1,2,4-triazolo[1,5-a] pyrimidine Metal Complexes: Antiparasitic Activity and Release Studies

Álvaro Martín-Montes [1,†], Sandra Jimenez-Falcao [2,†], Santiago Gómez-Ruiz [3], Clotilde Marín [1,*] and José M. Mendez-Arriaga [3,*]

Supplementary Information

1. UV SPECTRA

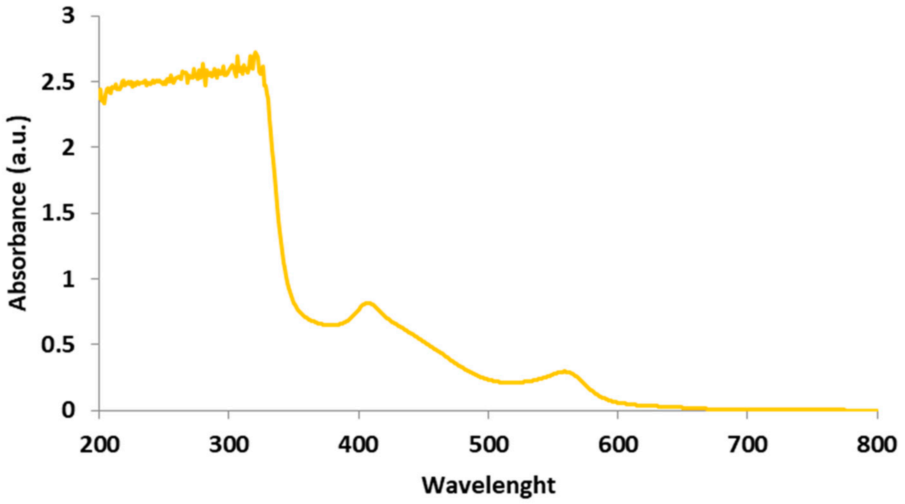

Figure S1. UV spectra complex **2** in MTL medium.

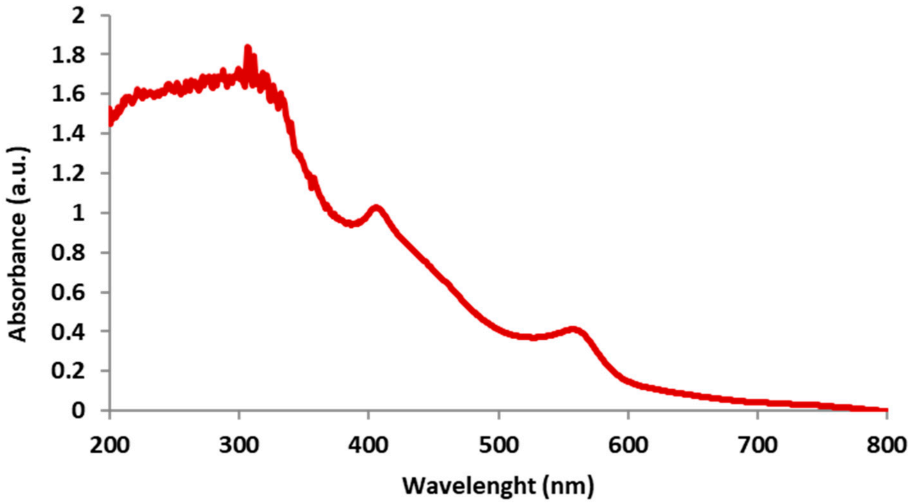

Figure S2. UV spectra complex **3** in MTL medium.

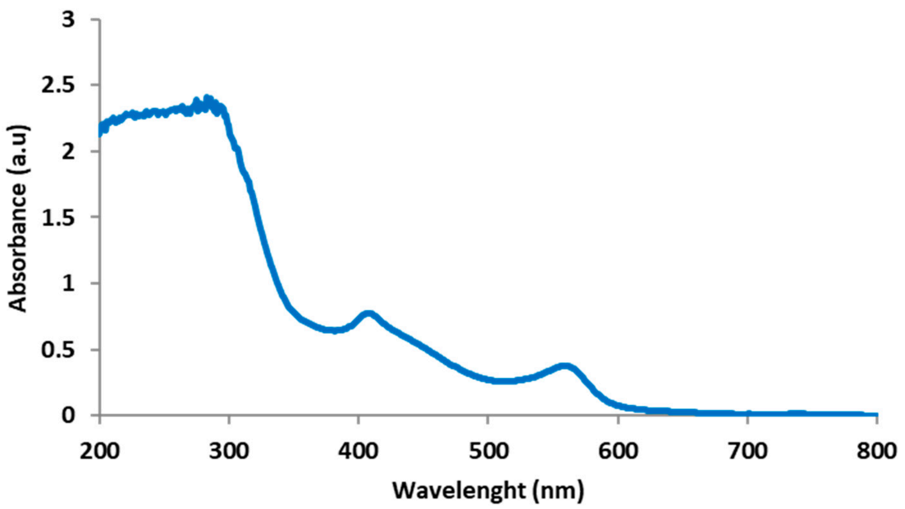

Figure S3. UV spectra complex **4** in MTL medium.
